# Epigenetic modifications of gut microbiota and their potential role in atherosclerosis

**DOI:** 10.3389/fphar.2025.1638240

**Published:** 2025-07-16

**Authors:** Shuang Guo, Junlai Zhao, Rongrong Zhu, Zhi Fan, Shibiao Liu, Weiwei Wu

**Affiliations:** Department of Vascular Surgery, Beijing Tsinghua Changgung Hospital, School of Clinical Medicine, Tsinghua Medicine, Tsinghua University, Beijing, China

**Keywords:** gut microbiota, epigenetic modification, atherosclerosis, DNA methylation, histone modification, non-coding RNA

## Abstract

Emerging evidence positions the gut microbiota as a pivotal regulator of host metabolism and immunity, particularly in atherosclerosis pathogenesis, with epigenetic mechanisms serving as fundamental mediators of gene expression control. This review systematically summarizes gut microbiome-driven epigenetic pathways, encompassing DNA methylation, histone modifications, non-coding RNA networks and their interplay with atherosclerosis-related pathological processes. We synthesize current evidence on microbiota-epigenome crosstalk, highlighting its potential mechanistic contributions to atherosclerotic plaque development.

## 1 Introduction

Atherosclerosis (AS) is an age-related disease characterized by fibrofatty lesions in the artery walls, the major cause of myocardial infarctions, strokes, and peripheral artery disease, leading to a major global burden of cardiovascular morbidity and mortality (Song et al., 2019). Well-known risk factors for AS include aging, hyperlipidemia, diabetes, and obesity, which cause endothelial dysfunction, lipoprotein retention, inflammatory cell recruitment, oxidative stress, foam cell formation, apoptosis and necrosis, vascular smooth muscle cell proliferation, matrix synthesis, calcification, angiogenesis and fibrous cap formation (Björkegren and Lusis, 2022).

Emerging evidence shows that gut microbiota and their metabolites are increasingly recognized as critical modulators in the progression of AS and other vascular diseases (Chen et al., 2023). Commensal microbes engage in dynamic crosstalk with intestinal epithelial cells (IECs) at the host-microbiota interface, orchestrating immune cell development through bidirectional signaling. Beyond local intestinal modulation, gut microbial metabolites and intestinal immune cells repertoire employ distinct transport pathways to disseminate systemically, establishing inter-organ communication networks that may contribute to the progression of vascular pathologies. Therefore, some experts describe the microbiota-artery axis, or gut-vascular axis, as a unified entity that contributes to these vascular conditions, making it a promising target for treatment (Zhang et al., 2023; Flori et al., 2024; Huang et al., 2025; Cook and Hogue, 2021). The underlying mechanisms sustaining this conceptual framework remain to be systematically elucidated.

**FIGURE 1:**
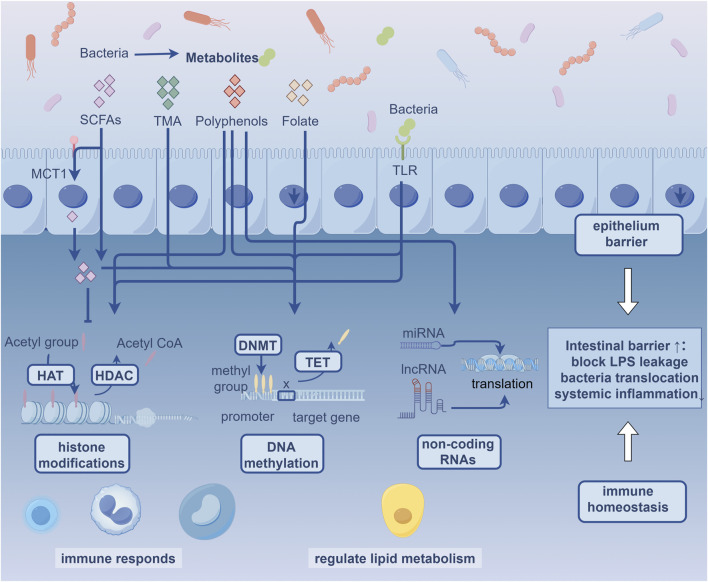
Gut Microbiota-Epigenome Potential Crosstalk in Atherosclerosis MCT1: Monocarboxylate transporter 1; SCFAs: Short Chain Fatty Acids; TMA: Trimethylamine; TLR: Toll-like receptors; HAT: Histone acetyltransferases; HDAC: Histone deacetylase; DNMT: DNA methyltransferases; TET: ten-eleven translocation.

Epigenetics investigates how endogenous and exogenous factors (diet, gut microbiota, medication, and environmental) modify gene expression without altering DNA sequences, including processes such as DNA methylation, histone modifications, and non-coding RNAs. The exogenous factors affect both the host’s epigenome and the composition and activity of gut microbiota, which in turn indirectly influence the host’s epigenome (Woo and Alenghat, 2022). In addition, epigenetic drive functional changes across heterogeneous vascular cell populations during atherogenesis progression. Recent years, the field of gut microbiota and epigenetics has gained attention among vascular clinicians and researchers. This review focuses on the latest findings regarding epigenetic modifications in gut microbiota and explores their potential roles in the development of AS (Figure 1).

## 2 Epigenetic modification

Genomic DNA is hierarchically packaged with histones into chromatin structures. In mammalian systems, DNA methylation primarily occurs as 5-methylcytosine (5 mC), an epigenetic modification catalyzed by DNA methyltransferases (DNMTs) through covalent methyl group addition to cytosine bases. While adenine methylation exists, 5 mC stands as the predominant epigenetic mark in mammals, serving as the principal regulator of gene silencing, chromatin structure, and genomic stability. This modification predominantly occurs at CpG islands, where it sterically hinders transcription factor binding and represses gene expression, particularly when localized near promoter regions (Zhao et al., 2020).

Transcriptionally active euchromatin maintains relaxed configurations permitting transcriptional machinery access, while condensed heterochromatin restricts DNA accessibility through histone-DNA complexes. Histone post-translational modifications constitute another primary mechanism for microbial regulation of host chromatin. Rather than targeting DNA directly, these covalent modifications, conjugated to lysine residues on histone tails, alter chromatin conformation and gene expression. Among >20 identified post-translational modifications classes, histone acetylation and methylation predominate in microbiota-host studies, dynamically regulated by opposing enzyme classes, such as histone acetyltransferases (HATs) *versus* deacetylases (HDACs), methyltransferases *versus* demethylases (Zhao et al., 2020).

Non-coding RNAs are functionally categorized based on nucleotide length. Long non-coding RNAs (lncRNAs, ≥200 nucleotides) modulate gene expression by serving as scaffolds for chromatin remodeling or by interacting with transcriptional regulators (Statello et al., 2021). In contrast, microRNAs (miRNAs, 18–25 nucleotides) mediate RNA interference by binding to mRNA untranslated regions, leading to translational repression or mRNA degradation (Yao et al., 2019).

## 3 Epigenetic modification of microbiota in atherosclerosis

The gut microbiota orchestrates host epigenetic reprogramming via three principal pathways: microbial-derived metabolites modulating substrate availability for DNA/histone modifications, regulation of epigenetic modifying enzyme expression and activity, and maintain intestinal epithelial barrier function that epigenetically coordinate transcriptional networks (Woo and Alenghat, 2022). These microbiota-epigenome interactions constitute a regulatory axis shaping host physiological plasticity. Further, epigenetic mechanisms maintain transcriptional reprogramming in host cells, sustaining altered gene expression patterns that continue beyond the removal of microbial stimuli.

### 3.1 Microbiota-derived metabolites

The gut microbiota generates bioactive metabolites that play roles as substrates, cofactors, or enzymatic modulators in chromatin modification, directly interfacing with host epigenetic regulation. Here, we summarize the relationship between metabolites produced by the gut microbiota and epigenetic modifications in atherosclerosis.

#### 3.1.1 Short chain fatty acids (SCFAs)

Gut microbiota-derived SCFAs (primarily acetate, propionate, and butyrate) are bioactive metabolites synthesized through fermentation of dietary fiber and degradation of proteins and aromatic compounds. SCFAs traverse cellular membranes via passive diffusion or active transport through monocarboxylate transporter 1. They can bind directly to intracellular HDACs and inhibit their activity. Short-chain lysine acylation is reversibly regulated by competing acyltransferases and deacylases. The acyltransferase superfamily (GCN5-related N-acetyltransferases, p300-CBP, MYST) catalyze site-specific short-chain acylation, while deacylase families (Zn^2+^-dependent HDACs and NAD^+^-dependent sirtuins) counteract these modifications.

Preclinical models and multi-omics profiling establish the causal protective role of SCFAs in AS progression by mapping vascular epigenetic remodeling. Butyrate and propionate promote Treg generation both by enhancing Foxp3 acetylation through HDAC inhibition and by serving as acyl-CoA donors for histone acetyltransferases (Arpaia et al., 2013; Thomas and Denu, 2021).

Butyrate orchestrates transcriptional programs across immune populations (macrophages, dendritic cells, Tregs) and intestinal epithelial cells by elevating histone acetylation and chromatin accessibility, driving a metabolic inflammatory resolution, and lipid metabolism (Schulthess et al., 2019; Furusawa et al., 2013; Grouls et al., 2022; Chang et al., 2014). Butyrate-mediated targeting of HDAC3/6 induces acetylation of non-histone proteins NF-κB subunit p65, altering its promoter binding capacity to attenuate pro-inflammatory transcriptional activation (Sarkar et al., 2023). In addition, butyrate exerts atheroprotective effects by epigenetically reprogramming vascular smooth muscle cell proliferation through chromatin remodeling-mediated cell cycle arrest. Mechanistically, it downregulates G1-specific cell cycle proteins while upregulating cdk inhibitors such as p15INK4b and p21Cip1 (Mathew et al., 2010).

Propionate may influence cardiovascular function via protein propionylation, with histone lysine propionylation (Kpr) as a potential key mechanism. Propionylation of histone H3 at Lys14 (H3K14pr) is predominantly enriched at promoters of highly transcriptionally activated genes, including those involved in fatty acid oxidation. Conversely, deficiency in H3K23pr mediated by BRPF1-KAT6 complexes contributes to cardiac anomalies. These findings suggest a potential role for histone propionylation in cardiovascular homeostasis, though the underlying mechanisms involving H3K14pr and H3K23pr in cardiovascular diseases require further investigation (Kebede et al., 2017).

Acetate induces histone H3 hyperacetylation, specifically activating lipogenic genes ACACA and FASN through increased H3K9, H3K27, and H3K56 acetylation at their promoters. This epigenetic regulation, mediated by acetyl-CoA synthetases ACSS1 and ACSS2, enhances *de novo* lipid synthesis in concert with acetate’s role as a fatty acid precursor (Gao et al., 2016). Macrophages and dendritic cells can sense butyrate in part through G-protein-coupled-receptors, that are correlates with increased global histone H3 acetylation (Ji et al., 2016).

SCFAs mediate cardioprotection through DNA methylation-dependent pathways. In type 2 diabetes patients, reduced Faecalibacterium prausnitzii (a key butyrate producer) correlates with hypermethylation of free fatty acid receptors promoter CpG sites (Chleilat et al., 2021). Acetate activates free fatty acid receptor two to stimulate leptin secretion in adipocytes, thereby regulating appetite and improving obesity (Chambers et al., 2015). Further, propionate induces specific DNA methylation patterns in the DAB adaptor protein 1 promoter, a diabetes target gene (Guo et al., 2022). Nevertheless, in high-fat-diet (HFD) models, acetate, propionate, butyrate suppress obesity-related leptin overexpression through downregulation DNMT1/3a/3b, reducing leptin promoter methylation (Lu et al., 2018). Although proposed mechanisms suggest SCFA-dependent HDAC inhibition modulates methyl-CpG-binding domain protein activity, this hypothesis remains speculative. Further *in vivo* tracer studies and chromatin profiling are warranted.

#### 3.1.2 Choline

Trimethylamine (TMA), a gut microbiota metabolite from dietary precursors (choline, phosphatidylcholine, and carnitine), serves as the direct precursor for trimethylamine-N-oxide (TMAO). Elevated TMAO levels predict and correlate with AS progression (Koeth et al., 2013). Mechanistically, *in vitro* studies reveal TMA exposure alters DNMT expression profiles—upregulating DNMT1 while suppressing DNMT3A (Shelp et al.). Mice harboring choline-metabolizing gut microbiota displayed reduced global DNA methylation and elevated inguinal adiposity under HFD conditions (Romano et al., 2017). It is hypothesized that bacterial choline metabolism depletes host methyl donors, reducing global DNA methylation and exacerbating HFD-induced metabolic dysregulation.

#### 3.1.3 Polyphenols

Polyphenols, predominantly present as glycosides, undergo colonic microbiota-mediated hydrolysis, demethylation, decarboxylation, dehydroxylation, and ring cleavage to form more bioactive metabolites. Current research primarily focuses on miRNA-related findings. Across both experimental models and human studies, polyphenols universally modulate miRNAs, predominantly affecting inflammation and lipid metabolism. Current evidence regarding miRNA-epigenetic regulation in atherosclerosis models remains limited. The few available studies report a correlation between overexpression of miR-181a, miR-106a, miR-20b and their target genes HIF1A/VEGFA, with this molecular signature aligning attenuated lesion progression. Notably, polyphenol-modulated anti-angiogenesis may operate through these miRNAs. A broad summary of polyphenols’ potential mechanistic roles in atherosclerosis pathogenesis follows.

Regarding inflammation regulation, resveratrol upregulates miR-663, a microRNA targeting multiple inflammatory genes, thereby suppressing endogenous activator protein-1 (AP-1) activity and attenuating lipopolysaccharide (LPS)-induced AP-1 activation *in vitro* (Tili et al., 2010). Supporting these mechanistic findings, a 12-month clinical trial in T2DM hypertensive patients demonstrated significant alterations in six inflammation-regulating miRNAs (miR-21; miR-181b; miR-663; miR-30c2; miR-155; miR-34a) within circulating immune cells following resveratrol supplementation (Tomé-Carneiro et al., 2013). In the context of lipid metabolism modulation, resveratrol inhibits *de novo* lipogenesis in adipose tissue *in vivo* through upregulation of miR-539-5p, which functions as the functional mediator of this metabolic suppression (Gracia et al., 2016). Additionally, multiple bioactive polyphenols including quercetin, isorhamnetin, olive oil hydroxytyrosol, propolis extracts, curcumin, açai berry compounds, red muscadine grape polyphenols, grape seed extract, tea catechins, and polydatin modulate inflammation, oxidative stress, and lipid metabolism through miRNA-mediated gene regulation (Koudoufio et al., 2020). Polyphenols downregulate pro-inflammatory cytokine expression via these miRNAs, indicating potential immunomodulatory benefits for atherosclerosis.

Beyond miRNA regulation, polyphenols modulate epigenetic pathways, including DNA hypermethylation, histone methylation, and acetylation, that may putatively influence AS progression. Raspberry polyphenol extract counteracts HFD suppression of H3K27 acetylation (H3K27Ac), thereby ameliorating obesity and insulin resistance (Fan et al., 2020). Quercetin and its derivative Q2 attenuate adipogenesis by epigenetically repressing key adipogenic genes *C/EBPα* and *PPARγ*. Chromatin immunoprecipitation revealed compound-induced chromatin remodeling at 5′regulatory regions, accompanied by increased levels of the repressive histone mark H3K9me2 and decreased levels of the activating mark H3K4me2 (Nettore et al., 2019).

#### 3.1.4 Folate

Nutrients critical for one-carbon metabolism, particularly folate, vitamin B6, and B12, regulate DNA methylation. Deficiencies in folate, vitamin B6, and B12 are associated with elevated homocysteine levels, which may contribute to systemic methyl donor insufficiency, reduced DNA methylation, endothelial dysfunction, and accelerated AS (Ma et al., 2017). In human studies, the duration of maternal folate supplementation prior to conception shows a significant positive correlation (*p* = 0.024) with offspring leptin gene CpG methylation levels, though the functional implications require further investigation (Pauwels et al., 2016). Notably, outcomes exhibit model- and dose-dependency. In obese murine models, high-dose prenatal folate alters offspring lipid metabolism while increasing DNA methylation at CpG sites within promoters of hepatic adipose triacylglyceride lipase (ATGL) and adipose lipoprotein lipase (LPL) genes (Yang et al., 2017). Conversely, rat studies reveal that combined dietary protein restriction and folate supplementation during pregnancy significantly reduces PPAR gene methylation in offspring liver, thereby attenuating metabolic disease risk (Lillycrop et al., 2005). Collectively, these findings highlight the context-dependent epigenetic effects on AS of folate interventions.

### 3.2 Epigenetic regulation of intestinal barrier

Emerging evidence establishes microbial-epigenomic regulation as a key mechanism governing function of IECs. IECs sense microbial components via Toll-like receptors (TLRs), with pioneering studies demonstrating microbiota-mediated epigenetic control of *Tlr4* expression (Takahashi et al., 2011). Microbiota epigenetically regulate intestinal development through DNA methylation and histone modifications. Germ-free mice exhibit reduced Tlr4 promoter methylation in colonic IECs, correlating with diminished gene expression and lipopolysaccharides hyporesponsiveness (Takahashi et al., 2009). IEC-specific *Dnmt1* deletion causes global hypomethylation, aberrant crypt formation, and stunted colon development (Elliott et al., 2015; Yu et al., 2015). HDAC3 mediates integration of microbiota-derived signals that maintain healthy intestinal homeostasis (Alenghat et al., 2013). Surface colonocytes metabolize butyrate for energy, thereby butyrate-exposed stem cells show elevated histone acetylation with impaired proliferation or repair (Donohoe et al., 2011). Preserving intestinal barrier integrity blocks lipopolysaccharides leakage, bacteria translocation and subsequent systemic inflammation. This contributes to atheroprotective effects.

Microbiota-host epigenomic interactions critically regulate immune homeostasis. For example (Song et al., 2019), macrophages/dendritic cells sense SCFAs via G protein-coupled receptors, elevating global H3 acetylation to enhance anti-inflammatory cytokine expression and Treg modulation (Björkegren and Lusis, 2022). SCFAs activate GPR43 signaling and inhibit HDACs, increasing *Foxp3* locus acetylation and expression to drive Treg differentiation. Additionally, microbiota directs Treg DNA methylation by upregulating Uhrf1—a DNMT1/HDAC1-binding adaptor protein (Obat et al., 2014). (Chen et al., 2023) Microbiota reduces *Cxcl16* expression by decreasing 5′CpG methylation, limiting invariant natural killer T cell development (Zhang et al., 2023). Microbial signals modulate intestinal innate lymphoid cell function through epigenomic reprogramming (Woo and Alenghat, 2017). Beyond preventing enteric inflammation and infections, intestinal immune homeostasis suppresses systemic low-grade inflammation and functions as an immunocyte reservoir that mitigates distant vascular pathologies. Current research has established that propionate facilitates the recirculation of colonic Tregs from the colon through colonic dLNs and circulating blood to the pathological vessels (Nakanishi et al., 2018; Yang et al., 2022). The translational applicability of this mechanism to AS requires rigorous validation.

## 4 Conclusion and prospect

The gut microbiota emerges as a master regulator of host epigenetics, forging a critical “gut-vascular axis” that mechanistically links gut microbiota with epigenetic regulation of vascular function. Through dynamic modulation of DNA methylation, histone modifications, and non-coding RNAs, microbiota-derived metabolites orchestrate vascular inflammation, lipid metabolism, and immune cell function. Butyrate and propionate suppress atherogenic pathways by modulating chromatin accessibility, driving *Foxp3*-mediated Treg differentiation while silencing NF-κB-dependent inflammation through p65 hyperacetylation. Simultaneously, intestinal barrier integrity, maintained by microbiota-epigenome crosstalk, prevents systemic endotoxemia and primes immunocytes for vascular recirculation. While current evidences indicated that microbiota and microbiota-derived metabolites participate in AS through epigenetic modifications, the pathological significance of these changes requires deeper mechanistic exploration.

Current understanding suggests that dietary patterns profoundly modulate the composition and function of the gut microbiota and its production of microbial metabolites. These microbiota-derived metabolites serve as substrates and regulators for epigenetic modifications. Consequently, dietary interventions targeting epigenetic mechanisms represent a viable therapeutic strategy, yet critical challenges persist. Key unresolved issues include precise mapping of metabolite gradients to vascular epigenetic signatures, the establishment of rigorous patient stratification criteria for clinical translation, and the definition of contraindications thresholds. Addressing these requires convergent experimental-computational methodologies. Spatially resolved metabolomics enables regional metabolite detection across intestinal niches, while complementary epigenetic co-localization techniques establish functional relationships between metabolite distributions and local epigenetic modifications. Single-cell multi-omics integration further resolves metabolic-epigenetic crosstalk at cellular resolution.

Moving forward, this nascent field requires substantial further investigation. Key research gaps include limited epigenetic studies on metabolites such as choline-derived TMAO, polyphenols, and folate in atherosclerosis pathogenesis, necessitating expanded mechanistic evidence. Future studies should delineate epigenetic divergence between early and late atherosclerosis phases, identifying key metabolite-driven switches that differentially modulate disease evolution. Research should prioritize identifying patient subpopulations with maximal predicted benefit from targeted microbial or epigenetic therapies. Consequently, addressing these knowledge gaps will be essential to translate microbiota-epigenetic insights into safe, effective therapeutic interventions for atherosclerosis.

## References

[B1] Alenghat T. Osborne L. C. Saenz S. A. Kobuley D. Ziegler C. G. K. Mullican S. E. (2013). Histone deacetylase 3 coordinates commensal-bacteria-dependent intestinal homeostasis. Nature 504 (7478), 153–157. 10.1038/nature12687 24185009 PMC3949438

[B2] Arpaia N. Campbell C. Fan X. Dikiy S. van der Veeken J. deRoos P. (2013). Metabolites produced by commensal bacteria promote peripheral regulatory T cell generation. Nature 504 (7480), 451–455. 10.1038/nature12726 24226773 PMC3869884

[B3] Björkegren J. L. M. Lusis A. J. (2022). Atherosclerosis: recent developments. Cell 185 (10), 1630–1645. 10.1016/j.cell.2022.04.004 35504280 PMC9119695

[B4] Chambers E. S. Morrison D. J. Frost G. (2015). Control of appetite and energy intake by SCFA: what are the potential underlying mechanisms? Proc. Nutr. Soc. 74 (3), 328–336. 10.1017/S0029665114001657 25497601

[B5] Chang P. V. Hao L. Offermanns S. Medzhitov R. (2014). The microbial metabolite butyrate regulates intestinal macrophage function *via* histone deacetylase inhibition. Proc. Natl. Acad. Sci. U. S. A. 111 (6), 2247–2252. 10.1073/pnas.1322269111 24390544 PMC3926023

[B6] Chen X. Zhang H. Ren S. Ding Y. Remex N. S. Bhuiyan M. S. (2023). Gut microbiota and microbiota-derived metabolites in cardiovascular diseases. Chin. Med. J. Engl. 136 (19), 2269–2284. 10.1097/CM9.0000000000002206 37442759 PMC10538883

[B7] Chleilat F. Schick A. Deleemans J. M. Reimer R. A. (2021). Paternal methyl donor supplementation in rats improves fertility, physiological outcomes, gut microbial signatures and epigenetic markers altered by high fat/high sucrose diet. Int. J. Mol. Sci. 22 (2), 689. 10.3390/ijms22020689 33445606 PMC7826956

[B8] Cook M. D. Hogue T. (2021). Exercise and the microbiome: mechanistic perspectives of the impact of exercise on the gut-vascular axis. mSystems 6 (4), e0065021–e0065021. 10.1128/mSystems.00650-21 34402640 PMC8407104

[B28] Donohoe DR Garge N Zhang X (2011). The microbiome and butyrate regulate energy metabolism and autophagy in the mammalian Colon: Cell Metab. Available online at: https://www.cell.com/cell-metabolism/fulltext/S1550-4131(11)00143-4?_returnURL=https%3A%2F%2Flinkinghub.elsevier.com%2Fretrieve%2Fpii%2FS1550413111001434%3Fshowall%3Dtrue 10.1016/j.cmet.2011.02.018 PMC3099420 21531334

[B9] Elliott E. N. Sheaffer K. L. Schug J. Stappenbeck T. S. Kaestner K. H. (2015). Dnmt1 is essential to maintain progenitors in the perinatal intestinal epithelium. Dev. Camb Engl. 142 (12), 2163–2172. 10.1242/dev.117341 PMC4483766 26023099

[B11] Fan R. You M. Toney A. M. Kim J. Giraud D. Xian Y. (2020). Red raspberry polyphenols attenuate high fat diet-driven activation of NLRP3 inflammasome and its paracrine suppression of adipogenesis *via* histone modifications. Mol. Nutr. Food Res. 64 (15), e1900995. 10.1002/mnfr.201900995 31786828 PMC9045478

[B12] Flori L. Benedetti G. Martelli A. Calderone V. (2024). Microbiota alterations associated with vascular diseases: postbiotics as a next-generation magic bullet for gut-vascular axis. Pharmacol. Res. 207, 107334. 10.1016/j.phrs.2024.107334 39103131

[B13] Furusawa Y. Obata Y. Fukuda S. Endo T. A. Nakato G. Takahashi D. (2013). Commensal microbe-derived butyrate induces the differentiation of colonic regulatory T cells. Nature 504 (7480), 446–450. 10.1038/nature12721 24226770

[B14] Gao X. Lin S. H. Ren F. Li J. T. Chen J. J. Yao C. B. (2016). Acetate functions as an epigenetic metabolite to promote lipid synthesis under hypoxia. Nat. Commun. 7, 11960. 10.1038/ncomms11960 27357947 PMC4931325

[B15] Gracia A. Miranda J. Fernández-Quintela A. Eseberri I. Garcia-Lacarte M. Milagro F. I. (2016). Involvement of miR-539-5p in the inhibition of *de novo* lipogenesis induced by resveratrol in white adipose tissue. Food Funct. 7 (3), 1680–1688. 10.1039/c5fo01090j 26952965

[B16] Grouls M. Janssen A. W. F. Duivenvoorde L. P. M. Hooiveld GJEJ Bouwmeester H. van der Zande M. (2022). Differential gene expression in iPSC-derived human intestinal epithelial cell layers following exposure to two concentrations of butyrate, propionate and acetate. Sci. Rep. 12, 13988. 10.1038/s41598-022-17296-8 35977967 PMC9385623

[B17] Guo W. Zhang Z. Li L. Liang X. Wu Y. Wang X. (2022). Gut microbiota induces DNA methylation *via* SCFAs predisposing obesity-prone individuals to diabetes. Pharmacol. Res. 182, 106355. 10.1016/j.phrs.2022.106355 35842183

[B18] HuangA.MaJ.ZhuH.QiY.JinY.ZhangM. (2025). Blood metabolites mediate causal inference studies on the effect of gut microbiota on the risk of vascular calcification. J. Adv. Res. 10.1016/j.jare.2025.03.038 40139524

[B19] Ji J. Shu D. Zheng M. Wang J. Luo C. Wang Y. (2016). Microbial metabolite butyrate facilitates M2 macrophage polarization and function. Sci. Rep. 6, 24838. 10.1038/srep24838 27094081 PMC4837405

[B20] Kebede A. F. Nieborak A. Shahidian L. Z. Le Gras S. Richter F. Gómez D. A. (2017). Histone propionylation is a mark of active chromatin. Nat. Struct. Mol. Biol. 24 (12), 1048–1056. 10.1038/nsmb.3490 29058708

[B21] Koeth R. A. Wang Z. Levison B. S. Buffa J. A. Org E. Sheehy B. T. (2013). Intestinal microbiota metabolism of L-carnitine, a nutrient in red meat, promotes atherosclerosis. Nat. Med. 19 (5), 576–585. 10.1038/nm.3145 23563705 PMC3650111

[B22] Koudoufio M. Desjardins Y. Feldman F. Spahis S. Delvin E. Levy E. (2020). Insight into polyphenol and gut microbiota crosstalk: are their metabolites the key to understand protective effects against metabolic disorders? Antioxidants 9 (10), 982. 10.3390/antiox9100982 33066106 PMC7601951

[B23] Lillycrop K. A. Phillips E. S. Jackson A. A. Hanson M. A. Burdge G. C. (2005). Dietary protein restriction of pregnant rats induces and folic acid supplementation prevents epigenetic modification of hepatic gene expression in the offspring. J. Nutr. 135 (6), 1382–1386. 10.1093/jn/135.6.1382 15930441

[B24] Lu Y. Fan C. Liang A. Fan X. Wang R. Li P. (2018). Effects of SCFA on the DNA methylation pattern of adiponectin and resistin in high-fat-diet-induced Obese Male mice. Br. J. Nutr. 120 (4), 385–392. 10.1017/S0007114518001526 29925443

[B25] Ma Y. Peng D. Liu C. Huang C. Luo J. (2017). Serum high concentrations of homocysteine and low levels of folic acid and vitamin B12 are significantly correlated with the categories of coronary artery diseases. BMC Cardiovasc Disord. 17, 37. 10.1186/s12872-017-0475-8 28109191 PMC5251223

[B27] Mathew O. P. Ranganna K. Yatsu F. M. (2010). Butyrate, an HDAC inhibitor, stimulates interplay between different posttranslational modifications of histone H3 and differently alters G1-specific cell cycle proteins in vascular smooth muscle cells. Biomed. Pharmacother. 64 (10), 733–740. 10.1016/j.biopha.2010.09.017 20970954 PMC2997917

[B29] Nakanishi Y. Ikebuchi R. Chtanova T. Kusumoto Y. Okuyama H. Moriya T. (2018). Regulatory T cells with superior immunosuppressive capacity emigrate from the inflamed Colon to draining lymph nodes. Mucosal Immunol. 11 (2), 437–448. 10.1038/mi.2017.64 28766553

[B30] Nettore I. C. Rocca C. Mancino G. Albano L. Amelio D. Grande F. (2019). Quercetin and its derivative Q2 modulate chromatin dynamics in adipogenesis and Q2 prevents obesity and metabolic disorders in rats. J. Nutr. Biochem. 69, 151–162. 10.1016/j.jnutbio.2019.03.019 31096072

[B31] Obata Y. Furusawa Y. Endo T. A. Sharif J. Takahashi D. Atarashi K. (2014). The epigenetic regulator Uhrf1 facilitates the proliferation and maturation of colonic regulatory T cells. Nat. Immunol. 15 (6), 571–579. 10.1038/ni.2886 24777532

[B32] Pauwels S. Ghosh M. Duca R. C. Bekaert B. Freson K. Huybrechts I. (2016). Dietary and supplemental maternal methyl-group donor intake and cord blood DNA methylation. Epigenetics 12 (1), 1–10. 10.1080/15592294.2016.1257450 27830979 PMC5270634

[B33] Romano K. A. Campo A. M. del Kasahara K. Chittim C. L. Vivas E. I. Amador-Noguez D. (2017). Metabolic, epigenetic, and transgenerational effects of gut bacterial choline consumption. Cell Host Microbe 22 (3), 279–290.e7. 10.1016/j.chom.2017.07.021 28844887 PMC5599363

[B34] Sarkar A. Mitra P. Lahiri A. Das T. Sarkar J. Paul S. (2023). Butyrate limits inflammatory macrophage niche in NASH. Cell Death Dis. 14 (5), 332. 10.1038/s41419-023-05853-6 37202387 PMC10195803

[B35] Schulthess J. Pandey S. Capitani M. Rue-Albrecht K. C. Arnold I. Franchini F. (2019). The short chain fatty acid butyrate imprints an antimicrobial program in macrophages. Immunity 50 (2), 432–445. 10.1016/j.immuni.2018.12.018 30683619 PMC6382411

[B36] Shelp G. V. Dong J. Orlov N. O. Malysheva O. V. Bender E. Shoveller A. K. (2024). Exposure to prenatal excess or imbalanced micronutrients leads to long‐term perturbations in one‐carbon metabolism, trimethylamine‐n‐oxide and DNA methylation in wistar rat offspring. Available online at: https://faseb.onlinelibrary.wiley.com/doi/10.1096/fj.202401018RR. 10.1096/fj.202401018RR 39212230

[B37] Song P. Rudan D. Zhu Y. Fowkes F. J. I. Rahimi K. Fowkes F. G. R. (2019). Global, regional, and national prevalence and risk factors for peripheral artery disease in 2015: an updated systematic review and analysis. Lancet Glob. Health 7 (8), e1020–e1030. 10.1016/S2214-109X(19)30255-4 31303293

[B38] Statello L. Guo C. J. Chen L. L. Huarte M. (2021). Gene regulation by long non-coding RNAs and its biological functions. Nat. Rev. Mol. Cell Biol. 22 (2), 96–118. 10.1038/s41580-020-00315-9 33353982 PMC7754182

[B10] Takahashi K Sugi Y Hosono A Kaminogawa S (2009). Epigenetic regulation of TLR4 gene expression in intestinal epithelial cells for the maintenance of intestinal Homeostasis1. J. Immunol. Am. Assoc. Immunol. 10.4049/jimmunol.0901271 19846881

[B39] Takahashi K. Sugi Y. Nakano K. Tsuda M. Kurihara K. Hosono A. (2011). Epigenetic control of the host gene by commensal bacteria in large intestinal epithelial cells. J. Biol. Chem. 286 (41), 35755–35762. 10.1074/jbc.M111.271007 21862578 PMC3195625

[B40] Thomas S. P. Denu J. M. (2021). Short-chain fatty acids activate acetyltransferase p300. eLife 10, e72171. 10.7554/eLife.72171 34677127 PMC8585482

[B41] Tili E. Michaille J. J. Adair B. Alder H. Limagne E. Taccioli C. (2010). Resveratrol decreases the levels of miR-155 by upregulating miR-663, a microRNA targeting JunB and JunD. Carcinogenesis 31 (9), 1561–1566. 10.1093/carcin/bgq143 20622002 PMC4647642

[B42] Tomé-Carneiro J. Larrosa M. Yáñez-Gascón M. J. Dávalos A. Gil-Zamorano J. Gonzálvez M. (2013). One-year supplementation with a grape extract containing resveratrol modulates inflammatory-related microRNAs and cytokines expression in peripheral blood mononuclear cells of type 2 diabetes and hypertensive patients with coronary artery disease. Pharmacol. Res. 72, 69–82. 10.1016/j.phrs.2013.03.011 23557933

[B43] Woo V. Alenghat T. (2017). Host-microbiota interactions: epigenomic regulation. Curr. Opin. Immunol. 44, 52–60. 10.1016/j.coi.2016.12.001 28103497 PMC5451311

[B44] Woo V. Alenghat T. (2022). Epigenetic regulation by gut microbiota. Gut Microbes 14 (1), 2022407. 10.1080/19490976.2021.2022407 35000562 PMC8744890

[B45] Yang F. Xia N. Guo S. Zhang J. Liao Y. Tang T. (2022). Propionate alleviates abdominal aortic aneurysm by modulating colonic regulatory T-Cell expansion and recirculation. JACC Basic Transl. Sci. 7 (9), 934–947. 10.1016/j.jacbts.2022.05.001 36317128 PMC9617133

[B46] Yang X. Huang Y. Sun C. Li J. (2017). Maternal prenatal folic acid supplementation programs offspring lipid metabolism by aberrant DNA methylation in hepatic ATGL and adipose LPL in rats. Nutrients 9 (9), 935. 10.3390/nu9090935 28846595 PMC5622695

[B47] Yao Q. Chen Y. Zhou X. (2019). The roles of microRNAs in epigenetic regulation. Curr. Opin. Chem. Biol. 51, 11–17. 10.1016/j.cbpa.2019.01.024 30825741

[B48] Yu D. H. Gadkari M. Zhou Q. Yu S. Gao N. Guan Y. (2015). Postnatal epigenetic regulation of intestinal stem cells requires DNA methylation and is guided by the microbiome. Genome Biol. 16, 211. 10.1186/s13059-015-0763-5 26420038 PMC4589031

[B49] Zhang Q. Zhang L. Chen C. Li P. Lu B. (2023). The gut microbiota-artery axis: a bridge between dietary lipids and atherosclerosis? Prog. Lipid Res. 89, 101209. 10.1016/j.plipres.2022.101209 36473673

[B26] Zhao LY Song J Liu Y (2020). Mapping the epigenetic modifications of DNA and RNA - PMC. Available online at: https://pmc.ncbi.nlm.nih.gov/articles/PMC7647981/ 10.1007/s13238-020-00733-7 PMC7647981 32440736

